# Slug-upregulated miR-221 promotes breast cancer progression through suppressing E-cadherin expression

**DOI:** 10.1038/srep25798

**Published:** 2016-05-13

**Authors:** Yi Pan, Jing Li, Yaqin Zhang, Nan Wang, Hongwei Liang, Yuan Liu, Chen-Yu Zhang, Ke Zen, Hongwei Gu

**Affiliations:** 1 State Key Laboratory of Pharmaceutical Biotechnology, Nanjing Advanced Institute for Life Sciences (NAILS), Nanjing University, 22 Hankou Road, Nanjing, Jiangsu 210093, China; 2 Jiangsu Engineering Research Center for microRNA Biology and Biotechnology, Nanjing, Jiangsu 210093, China; 3 Department of Biochemistry and Molecular Biology, School of Basic Medical Sciences, Nanjing Medical University, Nanjing, Jiangsu 210029, China; 4 Center for Inflammation, Immunity and Infection & Department of Biology, Georgia State University, Atlanta, GA30302, USA

## Abstract

It is generally regarded that E-cadherin is downregulated during tumorigenesis via Snail/Slug-mediated E-cadherin transcriptional reduction. However, this transcriptional suppressive mechanism cannot explain the failure of producing E-cadherin protein in metastatic breast cancer cells after overexpressing E-cadherin mRNA. Here we reveal a novel mechanism that E-cadherin is post-transcriptionally regulated by Slug-promoted miR-221, which serves as an additional blocker for E-cadherin expression in metastatic tumor cells. Profiling the predicted E-cadherin-targeting miRNAs in breast cancer tissues and cells showed that miR-221 was abundantly expressed in breast tumor and metastatic MDA-MB-231 cells and its level was significantly higher in breast tumor or MDA-MB-231 cells than in distal non-tumor tissue and low-metastatic MCF-7 cells, respectively. MiR-221, which level inversely correlated with E-cadherin level in breast cancer cells, targeted E-cadherin mRNA open reading frame (ORF) and suppressed E-cadherin protein expression. Depleting or increasing miR-221 level in breast cancer cells induced or decreased E-cadherin protein level, leading to suppressing or promoting tumor cell progression, respectively. Moreover, miR-221 was specifically upregulated by Slug but not Snail. TGF-β treatment enhanced Slug activity and thus increased miR-221 level in MCF-7 cells. In summary, our results provide the first evidence that Slug-upregulated miR-221 promotes breast cancer progression via reducing E-cadherin expression.

Understanding the mechanisms that govern tumor metastasis, a main reason of tumor-related mortality^1^, is a great challenge in cancer research^2^. Epithelial-mesenchymal transition (EMT) is a key step in the progression of tumors toward metastasis and invasion^3^. Cells that undergone EMT rapidly lose the cell–cell contacts, acquire mesenchymal properties and develop migratory and invasive capacity^4^. Although the EMT process is complex, the hallmark of EMT is the downregulation of E-cadherin, an essential adhesive molecule in the establishment of epithelial adhesion junction and a tight polarized cell layer^5^. Downregulation of E-cadherin expression has been found in carcinomas arising in various tissues^6^,^7^. In human breast cancer, loss of expression of E-cadherin affect the invasive or metastatic behavior of breast cancer cells and was associated with poorly differentiated tumors and poorer prognosis^8^,^9^. Previous studies revealed that E-box elements in the E-cadherin promoter played a critical negative regulatory role in E-cadherin gene transcription in breast cancer cell lines. Two zinc-finger transcription factors known to bind E-box elements, Slug and Snail, are potential repressors of E-cadherin transcription^5^,^10^,^11^,^12^. The correlation between the expression of Slug and the loss of E-cadherin transcripts was suggested by analyzing the expression patterns of Slug, Snail and E-cadherin in breast cancer cell lines^13^.

However, recent studies have shown that E-cadherin expression might be also modulated at a posttranscriptional level^1^. Although the level of E-cadherin expression is significantly decreased or even no during tumorigenesis, tumor cells still contain considerable amount of E-cadherin mRNA^14^,^15^. The disparity between E-cadherin protein and mRNA levels in metastatic tumor cells was also confirmed by our experiment of overexpressing E-cadherin protein in metastatic tumor cells, in which no E-cadherin protein was produced while E-cadherin mRNA was overexpressed (Zen *et al.*, unpublished). MicroRNAs (miRNAs), as a class of noncoding RNAs, that regulate gene expression at posttranscriptional level, resulting in either mRNA degradation or translational inhibition^16^,^17^. Accumulating evidence has demonstrated that miRNAs play a key role in the cellular processes of differentiation, proliferation, maturation and apoptosis^18^,^19^. Nagaoka *et al.* reported that knockdown of miR-200a in mammary glands prevented increases in E-cadherin mRNA expression and thus decreased E-cadherin signal^20^. Ma *et al.* reported that miR-9 could inhibit E-cadherin expression by binding to the 3′-UTR of E-cadherin mRNA^1^. However, in our E-cadherin reexpression experiment, only E-cadherin (ORF) region without 5′-UTR (contains transcription factor binding sites) and 3′-UTR (contains classical miRNA binding sites) has been cloned into expression vector, what caused that metastatic cancer cells fail to increase the protein level of E-cadherin with highly transcribed E-cadherin (ORF) mRNA level. This disparity between E-cadherin mRNA and protein level strongly argues that there is a previously unidentified mechanism to regulate E-cadherin expression at posttranscriptional level.

In the present study, we determined a novel miRNA-based regulatory mechanism for E-cadherin expression in metastatic breast cancer cell. We demonstrated that Slug specifically promoted miR-221 expression, which in turn, directly targeted the E-cadherin mRNA transcript, leading to reduction of E-cadherin protein level. Furthermore, both *in vitro* and *in vivo* results showed that Slug-promoted miR-221 enhanced the breast tumor progression through reducing E-cadherin protein expression.

## Results

### Posttranscriptional regulation of E-cadherin in breast tumor tissues and metastatic MDA-MB-231 cells

**Figure 1:**
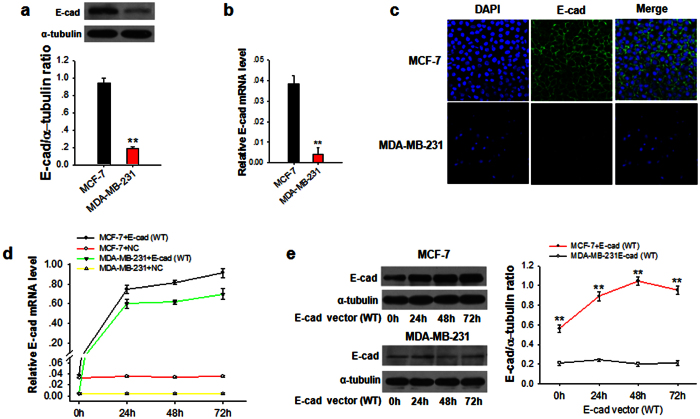
Restore E-cadherin expression in tumor cells using wild type (WT) E-cadherin-expressing vector, and compared the levels of E-cadherin protein and mRNA transcript at various time points. (**a**,**b**) E-cadherin protein levels (**a**) and mRNA levels (**b**) in MCF-7 and MDA-MB-231 cells. (**c**) Confocal immunofluorescent images of E-cadherin levels in MCF-7 cells and MDA-MB-231 cells. (**d**,**e**) E-cadherin mRNA levels (**d**) and protein levels (**e**) in MCF-7 and MDA-MB-231 cells transfected with WT E-cadherin-expressing vector (E-cad) for 0, 24, 48 or 72 h. Results are presented as the mean ± SEM (n = 3). *P < 0.05. **P < 0.01.

First, we compared the protein expression and mRNA level of E-cadherin in breast tumor tissues and distal normal tissue, as well as metastatic breast cancer MDA-MB-231 cells and non-metastatic MCF-7 cancer cells. In the experiment, 8 paired breast tumor tissue and distal normal tissue samples were collected and tested. As shown in Fig. 1a and Fig. S1, E-cadherin protein expression in metastatic MDA-MB-231 cells and breast tumor was significantly lower than that in normal tissue and MCF-7 cells, respectively. The levels of E-cadherin mRNA are also lower in MDA-MB-231and tumor tissue cells than in MCF-7 cells and normal tissue (Fig. 1b), respectively, which is in agreement with previous report that E-cadherin was suppressed at transcriptional level by Snail or Slug^5^,^13^. However, there was a certain disparity between the E-cadherin protein and mRNA in the tumor tissues, in which tumor cells displayed a certain amount of E-cadherin mRNA transcript but little or no E-cadherin protein (Fig. S1). In addition, immunofluorescence labeling also indicated that E-cadherin level of MDA-MB-231was less than that of MCF-7 cells (Fig. 1c). To further examine this disparity, we transfected MCF-7 cells and MDA-MB-231 cells with a wild type (WT) E-cadherin-expressing vector, and then compared the levels of E-cadherin protein and mRNA transcript at various time points. As expected, the levels of E-cadherin mRNA were markedly increased in both MCF-7 and MDA-MB-231 cells following the transfection (Fig. 1d). However, to our surprise, Western blot analysis (Fig. 1e) showed that, following overexpression of E-cadherin mRNA, the protein level of E-cadherin was only increased in MCF-7 cells but not MDA-MB-231 cells. This disparity between mRNA and protein levels of E-cadherin in MDA-MB-231 cells confirmed a posttranscriptional regulation for E-cadherin in metastatic MDA-MB-231 cells.

### Identification of miR-221 as a novel suppressor of E-cadherin expression

**Figure 2:**
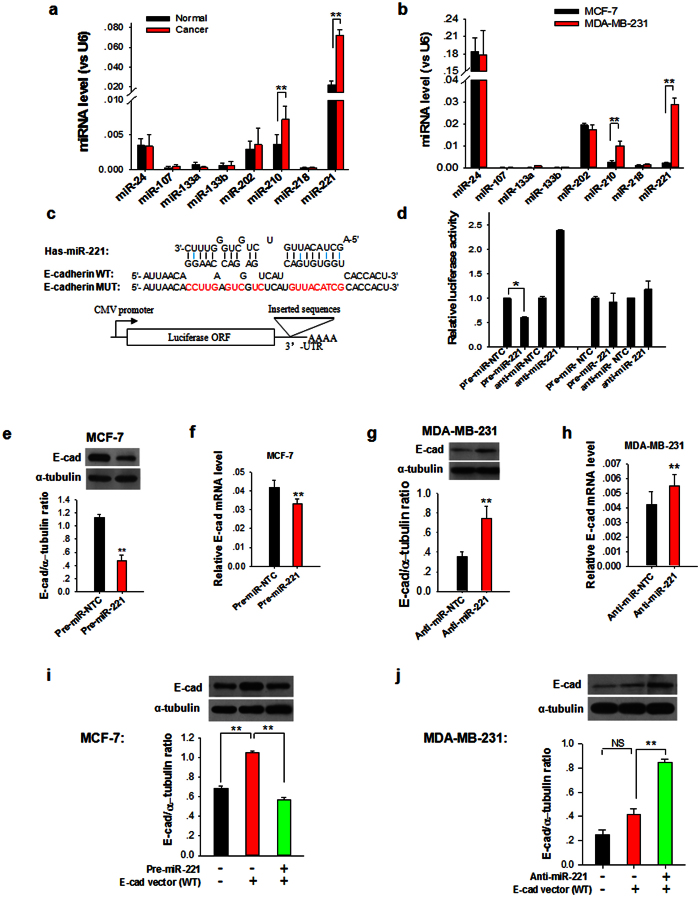
Posttranscriptional regulation of E-cadherin expression in breast cancer cells by miR-221. (**a**) Levels of all predicted miRNAs targeting E-cadherin ORF in paired breast cancer and normal tissue samples. (**b**) Levels of all predicted miRNAs targeting E-cadherin ORF in MCF-7 and MDA-MB-231 cells. (**c**) Schematic depiction of the Luciferase reporter consisting of full-length of E-cadherin ORF. The hypothetical duplexes formed by wild-type E-cadherin ORF and miR-221 were indicated. The mutated sites in E-cadherin MUT were marked in red. (**d**) Luciferase activity in MCF-7 cells transfected with either WT or Mut E-cadherin ORF plus pre-miR-221, anti-miR-221 or scrambled RNA oligonucleotides (control-pre-NTC). Cells were assayed using a luciferase assay kit 24 h post-transfection. (**e**,**f**) Overexpression of miR-221 in MCF-7 cells strongly reduced the expression of E-cadherin protein (**e**) and resulted in small but significant reduction in E-cadherin mRNA levels (**f**). (**g**,**h**) Knockdown of miR-221 level in MDA-MB-231 cells using anti-miR-221 ASO significantly induced E-cadherin protein expression (**g**) and resulted in E-cadherin mRNA slightly elevation (**h**). (**i**) E-cadherin protein levels in MCF-7 cells transfection with or without WT E-cadherin ORF and pre-miR-221. (**j**) E-cadherin protein levels in MDA-MB-231 cells transfected with or without WT E-cadherin ORF and anti-miR-221 ASO. Results are presented as the mean ± SEM (n = 3). NS, no significant difference, *P < 0.05. **P < 0.01.

Since the repression of mRNA transcripts by miRNAs is one of important posttranscriptional regulation, in which miRNAs block mammalian cell protein translation by base pairing to 3′-UTRs or ORF of target mRNA transcripts^21^, we postulated that miRNAs might be involved in regulating E-cadherin expression in MDA-MB-231 cells via targeting E-cadherin ORF. First, we used three computational algorithms, including TargetScan, miRanda and PicTar, to identify all oncomirs that potentially target E-cadherin ORF. With this approach, total of eight miRNAs, miR-24, miR-107, miR-133a, miR-133b, miR-202, miR-210, miR-218 and miR-221, were identified as candidate regulators of E-cadherin. The predicted interactions and the minimum free energy values of the hybridization between these miRNAs and the targeting sites within E-cadherin ORF are detailed in Supplementary Table S2. Next, we compared the levels of these potential E-cadherin-targeting miRNAs in breast tumor tissues and metastatic MDA-DB-231 cells to distal normal tissues and non-metastatic MCF-7 cells, respectively. As shown in Fig. 2a, by comparing 8 pairs of breast cancer tissues and distal non-cancerous tissues, we found that the levels of miR-221 and miR-210 were significantly higher in breast tumor tissues than in non-cancerous tissues. Moreover, the level of miR-221 was nearly 10 folds higher than that of miR-210. These results suggest that miR-221 may be the candidate miRNA responsible for E-cadherin posttranscriptional regulation in breast tumor tissues. A similar result was obtained by comparing metastatic MDA-MB-231 cells with non-metastatic MCF-7 cells (Fig. 2b). As shown, MDA-MB-231 cells displayed a significantly higher miR-221 level than MCF-7 cells. Level of miR-210 was also higher in MDA-MB-231 cells than in MCF-7 cells, but was less than one third of miR-221 level. Although miR-24 displayed the highest level in MDA-MB-231 cells, it was unlikely responsible for the differential expression of E-cadherin between MDA-MB-231 and MCF-7 cells because no difference of miR-24 level was found between two cell types.

Bioinformatics analysis predicted one putative miR-221 binding site within the ORF region of E-cadherin transcript (Fig. 2c). The minimum free energy value of the hybridization between miR-221 and E-cadherin was −26.1 kcal/mol, which is well within the range of genuine miRNA-target pairs. Furthermore, the miR-221 binding sequence in the E-cadherin ORF is highly conserved among primates such as Pan paniscus, Pan troglodytes, Rhinopithecus roxellana, etc. To test whether miR-221 can potentially bind E-cadherin mRNA, we cloned the fragment consisting of miR-221-binding sequence or mutated sequences without miR-221 binding into a luciferase reporter vector (pMIR-REPORT luciferase) (Fig. 2c). The luciferase reporter vectors were co-transfected into HEK-293T cells with Pre-miR-221 (synthetic RNA oligonucleotides mimicking miR-221 precursors) or control oligonucleotide (Pre-miR-NC). As shown in Fig. 2d, miR-221 was able to decrease the luciferase activity of the reporter vector containing miR-221 response element, while the reporter containing mutated sequences was not downregulated, confirming that miR-221 binds to the predicted sequence in E-cadherin mRNA.

The correlation between miR-221 and E-cadherin expression was further examined by evaluating E-cadherin expression in MCF-7 and MDA-MB-231 cells after overexpression or knockdown of miR-221. In these experiments, miR-221 overexpression was achieved by transfecting cells with Pre-miR-221, whereas miR-221 knockdown was achieved by transfecting cells with anti-miR-221 oligonucleotide (chemically modified antisense designed to specifically target mature miR-221). Clearly, the expression of E-cadherin protein was significantly inhibited by the introduction of miR-221 in MCF-7 cells (Fig. 2e), while anti-miR-221 significantly increased the E-cadherin protein level in MDA-MB-231 cells (Fig. 2g). To determine whether the expression of miR-221 affects the mRNA stability of E-cadherin, we designed primers for qRT-PCR to examine the expression of E-cadherin mRNA after transfection (Fig. 2f,h). This analysis revealed that stable expression of miR-221 in MCF-7 cells resulted in small but significant reduction in E-cadherin mRNA levels of 20% (Fig. 2f), and downregulation of miR-221 in MDA-MB-231 cells resulted in E-cadherin mRNA slightly elevation (Fig. 2h), which indicates that miR-221 regulate E-cadherin expression in breast cancer cells through canonical interaction as previously reported^22^. Consistent with previous studies that miRNA binding at ORF region only caused small, yet significant mRNA destabilization^23^. We also tested the effect of Pre-miR-221 and anti-miR-221 on E-cadherin expression in tumor cells that transfected with E-cadherin expressing vector (WT). As shown in Fig. 2i, increase of miR-221 in MCF-7 cells via Pre-miR-221 transfection remarkably reduced the E-cadherin expression induced by E-cadherin ORF expressing vector (WT). In contrast, although overexpression of E-cadherin ORF (WT) did not enhance E-cadherin protein expression in MDA-MB-231 cells, the E-cadherin protein level in E-cadherin expressing vector (WT)-transfected MDA-MB-231 cells was significantly increased after depleting cellular miR-221 by anti-miR-221 (Fig. 2j).

**Figure 3:**
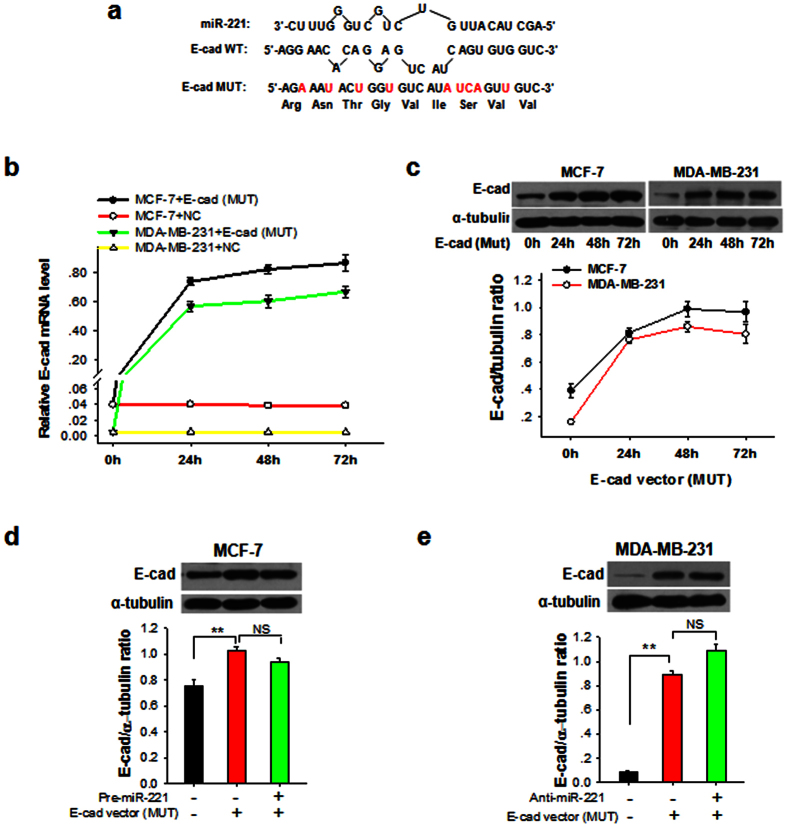
MDA-MB-231 cells can express functional E-cadherin that has the same amino acid sequence but not targeted by miR-221. (**a**) Construct of the vector encoding a mutated E-cadherin ORF (E-cad MUT) that has the same amino acids with WT E-cadherin but no miR-221 binding site. The mutated sites in E-cadherin MUT were marked in red. (**b**,**c**) E-cadherin mRNA levels (**b**) and protein levels (**c**) in MCF-7 and MDA-MB-231 cells transfected with E-cad MUT for 0, 24, 48 or 72 h. (**d**) E-cadherin protein levels in MCF-7 cells transfected with or without E-cad MUT and pre-miR-221. (**e**) E-cadherin protein levels in MDA-MB-231 cells transfected with or without E-cad MUT and anti-miR-221 ASO. Results are presented as the mean ± SEM (n = 3). NS, no significant difference, *P < 0.05. **P < 0.01.

To confirm that miR-221 regulates the E-cadherin gene by binding to the predicted binding site of ORF, we constructed a mutant E-cadherin overexpression vector (MUT), in which the miR-221 binding sequence was mutated without alteration of amino acid sequence (Fig. 3a). In the experiment, MCF-7 cells were co-transfected with Pre-miR-221 and E-cadherin vector (MUT), while MDA-MB-231 cells were co-transfected with anti-miR-221 and E-cadherin expression vector (MUT). As controls, MCF-7 cells were co-transfected with only Pre-miR-NC or Pre-miR-NC plus E-cadherin expression vector (MUT). MDA-MB-231 cells were co-transfected with anti-miR-NC or anti-miR-NC plus E-cadherin vector (MUT). We found that transfection with E-cadherin vector (MUT) strongly increased not only the level of E-cadherin mRNA transcript in both MCF-7 and MDA-MB-231 cells (Fig. 3b), but also E-cadherin protein level in both MCF-7 and MDA-MB-231 cells (Fig. 3c). Since E-cadherin vector (MUT) is not targeted by miR-221, the induction of E-cadherin protein by E-cadherin vector (MUT) should not be affected by miR-221. As expected, the enhanced expression of E-cadherin protein in MCF-7 (Fig. 3d) or MDA-MB-231 cells (Fig. 3e) by E-cadherin vector (MUT) transfection was not affected by overexpression of miR-221 or anti-miR-221, respectively.

### Specific upregulation of tumor cell miR-221 by Slug

**Figure 4:**
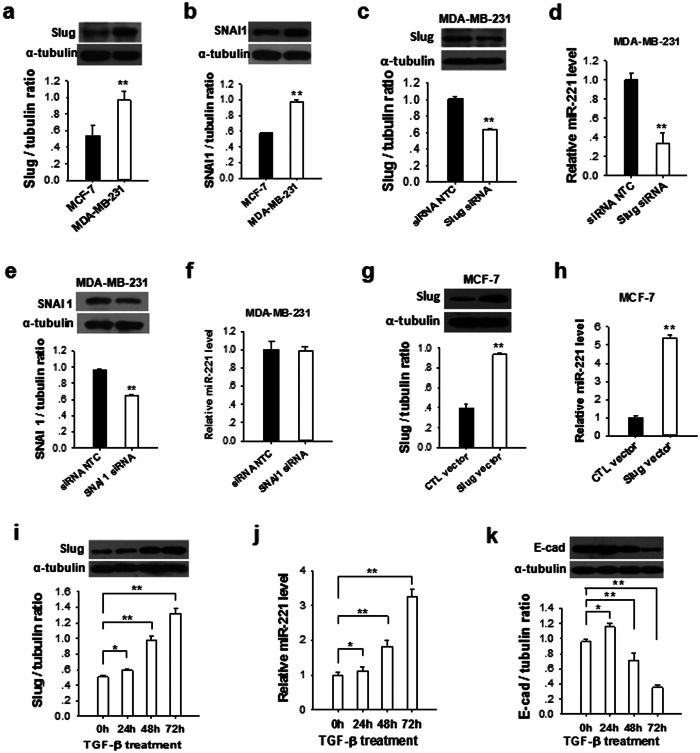
Upregulation of miR-221 in breast cancer cells by Slug but not Snail. (**a**,**b**) Slug protein levels (**a**) and SNAI1 protein levels (**b**) in MCF-7 and MDA-MB-231 cells. (**c**,**d**) Relative level of SNAI protein (**c**) and miR-221 levels (**d**) in MDA-MB-231 cells after transfection with SNAI1 siRNA or CTL-siRNA. (**e**,**f**) Relative level of SNAI protein (**e**) and miR-221 levels (**f**) in MDA-MB-231 cells after transfection with Slug siRNA or CTL-siRNA. (**g**,**h**) Relative level of Slug protein (**g**) and miR-221 levels (**h**) in MCF-7 cells after transfection with Slug-expressing vector (Slug vector) or control vector (CTL vector), (**i**) TGF-β (2 ng/ml) time-dependently increased the levels of Slug protein in MCF-7 cells. (**j**) TGF-β (2 ng/ml) time-dependently increased miR-221 level, (**k**) reduced E-cadherin protein expression in MCF-7 cells. Results are presented as the mean ± SEM (n = 3). *P < 0.05. **P < 0.01.

Snail family of zinc-finger transcriptional factors, including Slug and Snail (SNAI1), are master regulators of epithelial-mesenchymal transition (EMT)^24^,^25^. These transcriptional factors, particularly Slug, were strongly correlated with the suppression of E-cadherin transcripts^5^. To test whether Snail family transcriptional factors play a role in modulating miR-221 expression, we compared the levels of Slug and SNAI1 in MCF-7 and MDA-MB-231 cells. As shown in Fig. 4, a and b, the levels of Slug and SNAI1 in MDA-MB-231 cells were significantly higher than in MCF-7 cells. When knocked down Slug level in MDA-MB-231 cells via Slug siRNA (Fig. 4c), the cellular miR-221 level was significantly reduced (Fig. 4d). In contrast, when knocked down SNAI1 level in MDA-MB-231 cells via SNAI1 siRNA (Fig. 4e), the cellular miR-221 level was not affected (Fig. 4f). The results collectively suggest that Slug but not SNAI1 is involved in promoting miR-221 expression, which is in agreement with recent report that miR-221 is a Slug target gene and is transcriptionally regulated by Slug, silence of Slug decreased the level of miR-221 in MDA-MB-231 cells^26^. In support of this, when MCF-7 cells were transfected with Slug-expressing vector to increase Slug level (Fig. 4g), the cellular miR-221 expression was strongly enhanced (Fig. 4h).

Given that TGF-β has been reported to stimulate cell migration, invasion and metastasis of breast cancer cells by inducing the transcription factors Slug^27^, we next tested whether TGF-β1 treatment could promote miR-221 upregulation and E-cadherin downregulation in MCF-7 cells. In the experiment, MCF-7 cells were treated with 2 ng/ml TGF-β1 for 0, 24, 48 and 72 h, respectively. As shown in Fig. 4i, Slug protein level in MCF-7 cells was increased by TGF-β1 treatment in a time-dependent manner. In a similar fashion, the cellular level of miR-221 was time-dependently increased (Fig. 4j). In contrast, expression of E-cadherin was progressively decreased by TGF-β1 treatment (Fig. 4k).

### miR-221 enhances breast cancer cell migration and invasion via suppressing E-cadherin expression

**Figure 5:**
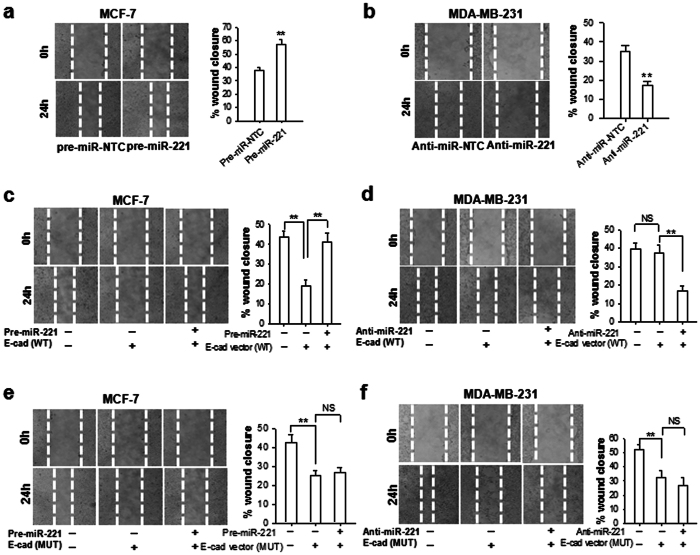
miR-221 promotes breast cancer cell migration via reducing E-cadherin. (**a**)Migration of MCF-7 cells transfected with pre-miR-221 or Pre-miR-CTL. (**b**) Migration of MDA-MB-231 cells transfected with anti-miR-221 ASO or anti-CTL ASO. (**c**) Migration of MCF-7 cells transfected with E-cad (WT) ORF, or pre-miR-221. (**d**) Migration of MDA-MB-231 cells transfected with E-cad (WT) ORF, or pre-miR-221. (**e**) Migration of MCF-7 cells transfected with E-cad (MUT) ORF, or anti-miR-221 ASO. (**f**) Migration of MDA-MB-231 cells transfected with E-cad (MUT) ORF, or anti-miR-221 ASO. Results are presented as the mean ± SEM (n = 3). NS, no significant difference, **P < 0.01.

As E-cadherin is a key adhesive molecule that prevents tumor cell metastasis, we determined whether targeting E-cadherin by miR-221 can promote breast cancer cell migration and invasion. The wound healing assays showed that the migration capacity of MCF-7 cells was significantly enhanced by overexpression of miR-221 (Fig. 5a). In contrast, the migration capacity of MDA-MB-231 cells was strongly reduced by depleting miR-221 via transfection with anti-miR-221 (Fig. 5b). Transfection with WT or MUT E-cadherin-expressing vectors both led to the inhibition of MCF-7 cell migration (Fig. 5c,e), whereas only transfection of MUT E-cadherin vector (Fig. 5d) but not WT E-cadherin vector (Fig. 5f) resulted in an inhibition of MDA-MB-231 cell migration. As expected, overexpression of miR-221 abolished the inhibitory effect of WT E-cadherin vector (Fig. 5c) but not MUT E-cadherin vector (Fig. 5e) on MCF-7 migration. In a similar fashion, depletion of miR-221 via transfection with anti-miR-221 significantly decreased the migration of MDA-MB-231 cells transfected with WT E-cadherin vector (Fig. 5d) but not MUT E-cadherin vector (Fig. 5f).

**Figure 6:**
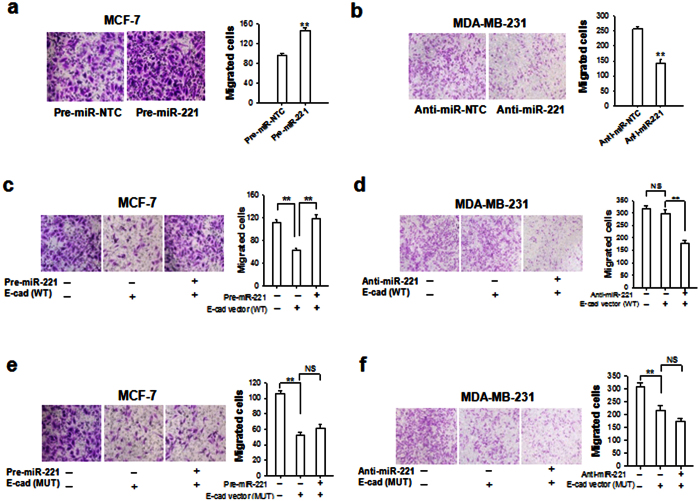
miR-221 promotes breast cancer cell invasion via reducing E-cadherin. (**a**) Invasion of MCF-7 cells transfected with pre-miR-221 or scrambled control oligonucleotide (pre-miR-NTC). (**b**) Invasion of MDA-MB-231 cells transfected with anti-miR-221 ASO or control ASO (anti-CTL ASO). (**c**) Invasion of MCF-7 cells transfected with or without E-cad (WT) ORF or pre-miR-221. (**d**) Invasion of MDA-MB-231 cells transfected with or without E-cad (WT) ORF, or anti-miR-221 ASO. (**e**) Invasion of MCF-7 cells transfected with or without E-cad (MUT) ORF, or pre-miR-221. (**f**) Invasion of MDA-MB-231 cells transfected with or without E-cad (MUT) ORF or anti-miR-221 ASO. Results are presented as the mean ± SEM (n = 3). NS, no significant difference, *P < 0.05. **P < 0.01.

Transwell invasion assay showed that overexpression of miR-221 in MCF-7 cells enhanced cell invasion (Fig. 6a), whereas depletion of miR-221 in MDA-MB-231 cells reduced cell invasion (Fig. 6b). When cells were transfected with WT or MUT E-cadherin expressing vectors, the invasion of MCF-7 cells was significantly reduced by both WT E-cadherin vector (Fig. 6c) and MUT E-cadherin vector (Fig. 6e). In contrast, the invasion of MDA-MB-231 cells was not affected by WT E-cadherin vector (Fig. 6d) but suppressed by MUT E-cadherin vector (Fig. 6f). When we overexpressed or depleted miR-221 in MCF-7 or MDA-MB-231 cells that were transfected with WT or MUT E-cadherin vectors, we found that overexpression of miR-221 in MCF-7 cells only abolished the inhibitory effect of WT E-cadherin vector (Fig. 6c) but not MUT E-cadherin vector (Fig. 6e) on cell invasion. Similarly, depletion of miR-221 significantly reduced the invasion of MDA-MB-231 cells transfected with WT E-cadherin vector (Fig. 6d) but not MUT E-cadherin vectors (Fig. 6f). Taken together, these results demonstrated that miR-221 promotes breast cancer cell migration by suppressing E-cadherin expression.

### Targeting E-cadherin by miR-221 promotes breast tumor cell metastasis in mouse model

To evaluated the role of miR-221 and E-cadherin in mediating breast cancer metastasis in mouse model, we first designed and generated four types of modified MDA-MB-231 cell lines: cells infected with control lentivirus (control), cells stably transfected with WT E-cadherin (E-cad (WT)), cells stably transfected with WT E-cadherin combined with anti-miR-221 (E-cad (WT) plus anti-miR-221), and cells stably transfected with MUT E-cadherin (E-cad (MUT)). The qRT-PCR assay and Western blot analysis confirmed that the designed four cell lines were successfully produced (Supplemental Fig. S2a–c). *In vitro* wound healing and Transwell invasion assays showed that two modified MDA-MB-231 cell lines, E-cad (MUT) and E-cad (WT) plus anti-miR-221, displayed a decreased migration and invasion compared to control MDA-MB-231 cells and MDA-MB-231 cells stably transfected with E-cad (WT) (Supplemental Fig. S2d,e).

**Figure 7:**
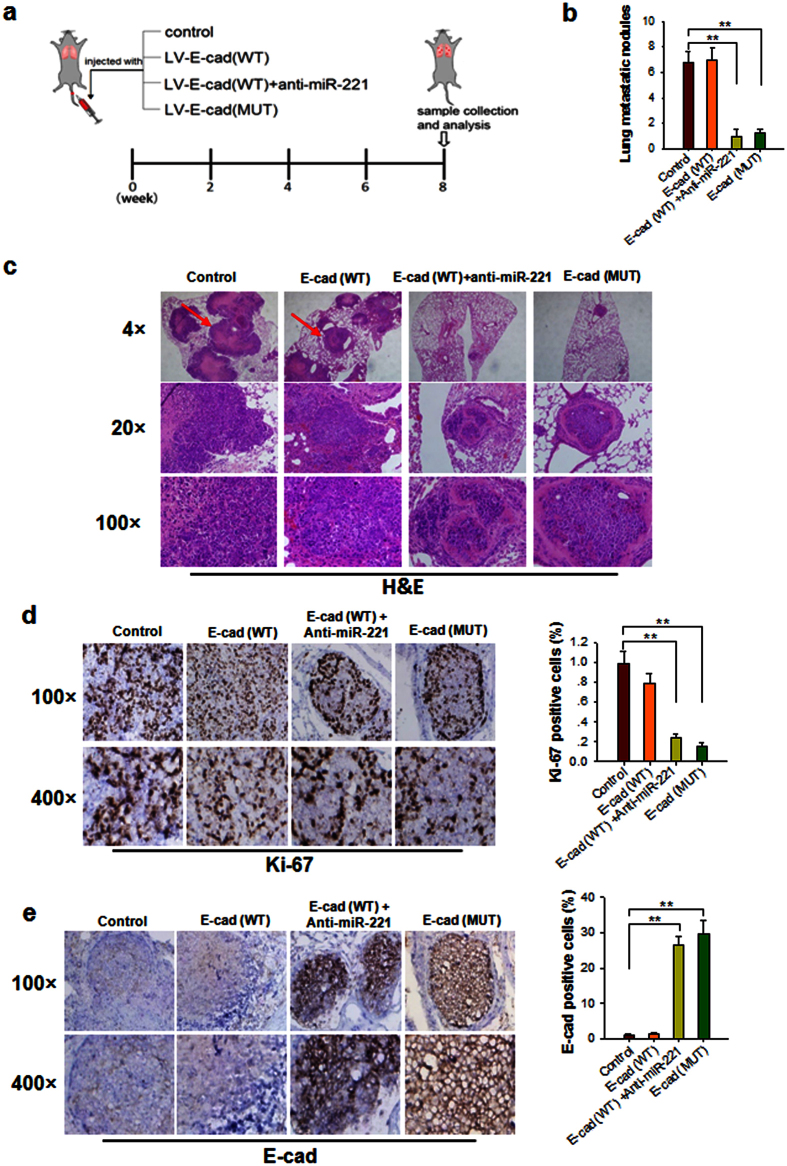
E-cadherin inhibits the lung colonization of MDA-MB-231 cells. (**a**) Experimental design. Immunocompromised mice were injected through tail vein with control MDA-MB-231 cells or MDA-MB-231 cells that were stably transfected with E-cad (WT), E-cad (WT) plus anti-miR-221 or E-cad (Mut). After 8 weeks, mice were sacrificed and lungs were extracted. (**b**) the numbers of tumor nodules in the lungs. Results were derived from five mice in each group. (**c–e**) Mouse lungs were subjected to H&E staining (**c**) and immunohistochemical staining for Ki-67 (**d**) and E-cadherin (**e**), respectively. Left panel: representative image; right panel: quantitative analysis of images. Results are presented as the mean ± SEM (n > 3). **P < 0.01.

Subsequently, we injected these four modified MDA-MB-231 cell lines into female nude mice (6 weeks, 22–24 g) through the tail vein (Fig. 7a). After 8 weeks, mice were killed and whole lung tissues were harvested, and the numbers of macroscopically visible tumor nodules on the lung surface were counted. Mouse lung tissues were also fixed in 10% formalin and embedded in paraffin, sectioned and subjected to H&E staining for evaluating tumor metastasis or immunohistochemical staining for detecting Ki-67 and E-cadherin expression. As shown in Fig. 7b, metastasis of control or E-cad (WT) MDA-MB-231 cells to mouse lungs was clearly observed, whereas E-cad (WT) plus anti-miR-221 or E-cad (MUT) MDA-MB-231 cells showed significantly less lung metastatic activity. The mice injected with E-cad (WT) plus anti-miR-221 or E-cad (MUT) MDA-MB-231 cells had a significantly decreased number of lung nodules compared with those injected with control or E-cad (WT) MDA-MB-231 cells.

HE staining also showed a significant difference of tumor number and growth in mouse lungs among these modified MDA-MB-231 cells (Fig. 7c). In mice injected with control or E-cad (WT) MDA-MB-231 cells, 5–10 different sized tumors with clear boundaries (arrows) were found in the lungs. The tumor cells were arranged in a prominent nesting pattern and some tumor tissues had fused. Numerous abnormal large necrosis areas could be observed in the center of tumor mass. In contrast, in mice injected with E-cad (WT) plus anti-miR-221 or E-cad (MUT) MDA-MB-231 cells, only 1–2 smaller tumor masses were scattered in the lungs, and few small necrosis spots were found in the center of tumor mass. Immunohistochemical studies using Ki-67 staining further showed a significantly decrease of tumor cell proliferation in mouse lungs from the mouse groups implanted with E-cad (WT) plus anti-miR-221 or E-cad (MUT) MDA-MB-231 cells (Fig. 7d). Moreover, E-cadherin labeling also revealed that the mouse groups implanted with E-cad (WT) plus anti-miR-221 or E-cad (MUT) MDA-MB-231 cells had higher level of E-cadherin than the group implanted with control or E-cad (WT) MDA-MB-231 cells (Fig. 7e). These results validated the role of miR-221 in promoting breast tumor metastasis in mice through suppressing E-cadherin expression.

## Discussion

Downregulation of E-cadherin expression is a leading event in the progression of various tumors into the metastatic cascade^2^,^28^,^29^. Different molecular mechanisms that govern the E-cadherin downregulation during tumorigenesis have been proposed and demonstrated^6^,^10^,^12^,^30^. In the present study, we show a novel mechanism to silence E-cadherin expression in metastatic tumor cells, in which, miR-221, upregulated by Slug, targets the ORF of E-cadherin mRNA transcript and suppresses E-cadherin expression in metastatic tumor cells.

Compared to healthy cells, tumor cells often displayed abnormal expression profile of miRNAs^19^,^31^. Given their opposing function in tumorigenesis, certain miRNAs were also termed as oncomir or tumor suppressors. In general, oncomirs promote tumor progression and their expression levels are significantly elevated in tumor cells and tissues, whereas tumor suppressors inhibit tumor progression and their expression levels are downregulated during tumorigenesis. Here we employed several strategies to search the oncomir that can directly target E-cadherin mRNA transcript, particularly the ORF of E-cadherin, and several pieces of evidence supported miR-221 as the one that is responsible for the posttranscriptional regulation of E-cadherin in metastatic tumor cells. First, we compared the expression levels of all oncomirs that can target E-cadherin ORF in breast cancer tissues or metastatic MDA-MB-231 cells with those in distal non-tumor tissues or non-metastatic MCF-7 cells, respectively. Among miR-24, miR-107, miR-133a, miR-133b, miR-202, miR-210, miR-218 and miR-221, we found that only miR-221 had not only a high abundance in breast cancer tissues or MDA-MB-231 cells but also displayed the highest fold-change when compared breast cancer tissues or MDA-MB-231 cells to distal non-tumor tissues or MCF-7 cells. More important, miR-221 level and E-cadherin protein level in breast cancer tissues or MDA-MB-231 cells were inversely correlated. This inversed correlation between miR-221 and E-cadherin protein was further shown by treating MCF-7 cells with TGF-β (Fig. 4I,k), in which E-cadherin protein level in MCF-7 cells was decreased but miR-221 level was increased. Second, luciferase reporter assay confirmed that the pairing of miR-221 with the seeded sequence on the E-cadherin ORF with the minimum free energy value of –26.1 kcal/mol (Supplementary Table S2). The miR-221 binding sequence in the E-cadherin ORF is also highly conserved. It has been shown that miR-222, an oncomir encoded as a cluster with miR-221 on chromosome X, is also overexpressed in many types of cancer, including thyroid carcinoma, glioblastoma, prostate carcinoma, bladder cancer, pancreatic cancer, hepatocellular carcinoma, acute myeloid leukemia and diffuse large B cell lymphoma^31^,^32^. However, no binding site of miR-222 on E-cadherin ORF was predicted in our study and luciferase reporter assay confirmed a poor binding between miR-222 and E-cadherin ORF (data not shown). Finally, we found that miR-221 was upregulated by transcriptional factor Slug but not Snail (Fig. 4). In agreement with the role of Slug and miR-221 in tumor metastasis, both Slug and miR-221 were upregulated in MDA-MB-231 cells not MCF-7 cells (Fig. 4). This finding provides the first evidence that miR-221 is specifically modulated by Slug. Previous study by Kim *et al.* showed that target of mdm2 by miR-221 could prevent the degradation of the Slug^33^. Combining this result with our finding, it suggests that miR-221 and Slug may form a vicious cycle in promoting tumor metastasis. In this cycle, Slug promotes miR-221 expression, and in turn, upregulation of miR-221 prevents the degradation of Slug and consequently upregulates Slug. Furthermore, in line with Slug-promoting miR-221 in metastatic tumor cells, our results also showed that both Slug and miR-221 were upregulate by TGF-β (Fig. 4I,j). Although the mechanism remains unclear, our data strongly argue the role of Slug-promoted miR-221 in tumorigenesis induced by TGF-β.

Since E-cadherin is an important adhesive molecule preventing cell EMT and tumorigenesis^34^, restoration of E-cadherin expression may serve as an efficient strategy in anti-tumor gene therapy. For example, restoring the re-expression of the intracellular domain of E-cadherin in E-cadherin–deficient MDA-MB-231 cells promoted the transition of tumor cells from a motile phenotype to a sessile cellular phenotype, suggesting that E-cadherin is potential factor for the treatment of the breast cancer^35^. However, due to the strong posttranscriptional modulation of E-cadherin in metastatic breast tumor cells, it is difficult to produce E-cadherin protein in high-grade malignant breast cancer cells through overexpression of E-cadherin mRNA transcript. Identification of the role of Slug-promoted miR-221 in suppressing E-cadherin protein expression thus extends our understanding of the regulatory mechanism of E-cadherin expression and also provides a new approach for restoring E-cadherin protein level in metastatic tumor cells and suppressing tumor progression.

**Figure 8:**
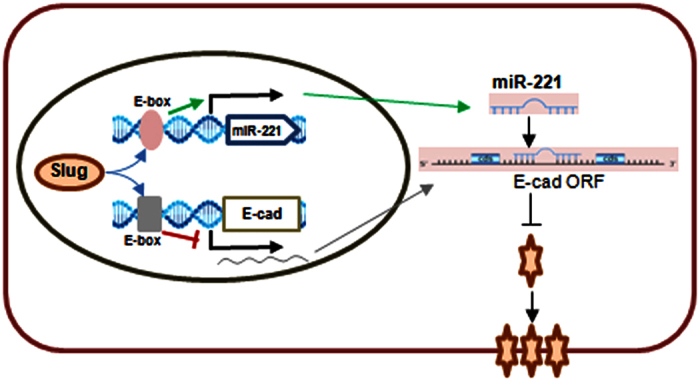
A working model that illustrates the mechanism by which Slug suppresses E-cadherin at both transcriptional and posttranscriptional level.

Although the association of upregulated Slug with reduced E-cadherin expression has been shown in previous studies^36^,^37^, the mechanism that governs the downregulation of E-cadherin by Slug is not completely understood. Bolós reported that Slug behaved as a transcriptional repressor of E-cadherin in a way similar to Snail, E12/E47, ZEB-1 and SIP-1^10^,^11^,^30^,^38^,^39^, through an interaction with the proximal E-boxes of E-cadherin promoter^12^. However, other studies did not support such repressive mode for Slug. For example, overexpression of Slug in rat bladder carcinoma cells did not repress E-cadherin^40^. Analysis of mouse epidermal keratinocyte cell lines also failed to show any correlation between E-cadherin and Slug expression profiles^12^. Our finding here provides a new miRNA-based mechanism to explain the downregulation of E-cadherin by Slug. As shown in the working model (Fig. 8), instead of suppressing E-cadherin transcription via direct binding to the E-boxes of E-cadherin promoter, Slug upregulates miR-221 expression, which in turn, suppresses the protein production of E-cadherin at posttranscriptional level.

## Materials and Methods

### Reagents, cells and antibodies

The human breast cancer cell lines MCF-7 and MDA-MB-231 was purchased from the Shanghai Institute of Cell Biology, Chinese Academy of Sciences (Shanghai, China). TGF-β1 was purchased from R&D Systems (Minneapolis, MN). Antibodies were from various sources: anti-E-cadherin antibody (BD Biosciences, Bedford, MA), anti-SLUG antibody (Cell Signaling, Beverley, MA), anti-SNAIL1 and anti-α-tubulin antibodies (Santa Cruz Biotechnology, Santa Cruz, CA). Antibody used for immunofluorescence staining in MCF-7 and MDA-MB-231 cells was anti-E-cadherin (BD Biosciences, Bedford, MA).

### Human tissue

Eight-paired breast tumor tissues and adjacent cancer tissues were collected from patients undergoing a surgical procedure at the Affiliated People’s Hospital of Jiangsu University (Zhenjiang, China). Both tumors and noncancerous tissues were confirmed histologically. The pathological type of each cancer was determined to be infiltrating ductal carcinoma. All of the patients provided written consent, and the Ethics Committee from Nanjing University approved all aspects of this study, and the methods were carried out in accordance with the approved guidelines. Tissue fragments were immediately frozen in liquid nitrogen at the time of surgery and stored at −80 °C. The clinical features of the patients are listed in Supplementary Table S1.

### RNA isolation and quantitative RT-PCR

A TaqMan probe–based qRT-PCR assay was performed to quantitative determination of serum miRNAs according to the manufacturer’s instructions (7300 Sequence Detection System, Applied Biosystems) as described previously^41^. U6 snRNA was used as an internal control, and the relative amount of miRNA normalized to U6 was calculated with the equation 2^−ΔΔCT^, in which ΔΔC_T_ = (C_T miRNA_ − C_T U6_)_target_ − (C_T miRNA_ − C_T U6_)_control_. To quantify E-cadherin and β-actin mRNA, Real-time PCR was performed according to previous publications^42^. The sequences of the primers were as follows: E-cadherin (sense): 5′-GAGTGCCAACTGGACCA TTCAGTA-3′; E-cadherin (antisense): 5′-AGTCACCCACCTCTAAGGCCATC-3′; β-actin (sense): 5′-AGGGAAATCGTGCGTGAC-3′; β-actin (antisense): 5′-CGCTCATTGCCGATAGTG-3′. T he C_T_ values were determined by setting a fixed threshold. The amount of E-cadherin mRNA was normalized to β-actin.

### miRNA overexpression or knockdown

miRNA overexpression was achieved by transfecting cells with miRNA mimics, whereas knockdown was achieved by transfecting cells with a miRNA inhibitor according to previous publications^42^. miRNA overexpression was achieved by transfecting cells with miRNA mimics (a synthetic RNA oligonucleotide duplex mimicking miRNA precursor), whereas knockdown was achieved by transfecting cells with a miRNA inhibitor (a chemically modified single-stranded antisense oligonucleotide designed to specifically absorb target miRNA). Synthetic RNA molecules, including pre-miR-221, anti-miR-221 and scrambled negative control RNA (pre-miR-control and anti-miR-control), were purchased from GenePharma (Shanghai, China). MCF-7 or MDA-MB-231 cells were seeded in 6-well plates and transfected with Lipofectamine 2000 (Invitrogen) on the following day when the cells were approximately 70% confluent. Cells were transfected with 50 pmol pre-miR-221 or anti-miR-221 to overexpress or knockdown cellular miR-221. After 6 h, the medium was changed to DMEM or L-15 medium that was supplemented with 2% fetal bovine serum. The cells were harvested 24 or 48 h posttransfection for RNA and protein analysis.

### Plasmid construction, siRNA interference assay and luciferase reporter assay

The mammalian expression plasmids designed to specially express ORF of human E-cadherin with either wild-type (WT) was purchased from GeneCopoeia and mutant (binding sites that interact with miR-221 were mutated) form of ORF was contructed in this study. The siRNA (sense: 5′-TTAGAGTCCTGCAGCTCGCdTdT-3′ and anti-sense: 5′-GCGAGCTGCAGGACTCTAAdTdT-3′) targeting human SLUG and (sense: 5′-CCCUGGUUGCUUCAAGGACACAUUAdTdT-3′, anti-sense: 5′-UAAUGUGUCCUUGAAGCAACCAGGGdTdT-3′) were synthesized by GenePharma. Overexpression plasmid or siRNA were transfected cells using Lipofectamine 2000 (Invitrogen) according to the manufacturer’s instructions. To test the direct binding of miR-221 to the target gene E-cadherin, a luciferase reporter assay was performed according to previous publications^43^.

### Western blotting and immunofluorescence

E-cadherin, SLUG and SNAIL1 protein levels were quantified by western blot analysis of whole-cell extracts using antibodies. Normalization was performed by blotting the same samples with an antibody against α-tubulin. Protein bands were analyzed using Bandscan software (Image J). Immunofluorescence analysis was performed according to previous publications^43^. Cells were grown on coverslips, fixed in 4% paraformaldehyde in PBS for 10 min, washed with PBS, and cooled with 100% methanol at −20 °C for 20 min. Thereafter, cells were washed with PBS and permeabilized with 0.1% Triton X-100 for 10 min. After blocking with Dako blocking solution, primary antibody (anti-E-cadherin, 1:100) was added and incubated at 4 °C overnight. For the secondary antibody, FITC-conjugated donkey anti-mouse (1:100) was applied for 60 min at room temperature in the dark followed by a PBS wash. DAPI (Sigma, St. Louis, MO) was used as a nuclear counterstain for 30 min. Following a final wash with PBS and addition of Slow Fate equilibration buffer (Molecular Probes, Eugene, OR), slides were mounted with 10μl Slow Fade (Molecular Probes) and observed under confocal microscopy (FV1000; Olympus, Tokyo). The pictures were taken under the following conditions: Objective Lens: PLAPON 40 × O NA: 1.42; Scan Mode: XY; Excitation Wavelength: 405 nm for DAPI and 488 nm for FITC; Image Size: 1024 × 1024 Pixel.

### Cell migration assay

The migrations of MCF-7 and MDA-MB-231 cells transfected with different miRNAs or plasmids were tested in a Transwell Boyden Chamber (6.5 mm, Costar, Cambridge, MA). The polycarbonate membranes (8-μm pore size) on the bottom of the upper compartment of the transwells were coated with 1% human fibronectin (R&D systems). Cells were harvested 48 hours later after transfection, and suspended in FBS-free DMEM or L-15 culture medium. Then cells were added to the upper chamber (4 × 104 cells/well). At the same time, 0.5 ml of DMEM or L-15 with 10% FBS was added to the lower compartment, and the plates were incubated for 8–12 hours in a 5% CO_2_ atmosphere saturated with H_2_O. After incubation, cells that had entered the lower surface of the filter membrane were fixed with 4% paraformaldehyde for 25 minutes at room temperature, washed 3 times with distilled water, and stained with 0.1% crystal violet in 0.1 M borate and 2% ethanol for 15 minutes at room temperature. Cells remaining on the upper surface of the membranes were scraped off gently with a cotton swap. The upper surfaces with migrant cells were captured 5–6 fields per chamber by a photomicroscope (BX51, Olympus, Japan), and the number of cells were counted blindly.

### Wound healing assay

Scratch wound healing assay was performed to assess cell migration. In brief, Cells were seeded into fibronectin coated 12-well microtiter plates at densities of 120000 cells per well and transfected with different miRNAs or plasmids the following day. Cells were wounded with a plastic pipette tip and the remaining cells were washed twice with fresh medium to remove cell debris the next day. After 16–24 hours incubation, the migrant cells at the wound front were photographed with a microscope. Wound closure was calculated and expressed as a percentage of the initial wound width.

### Construction of stable cell lines

Viruses produced using the vectors encoding E-cadherin (WT or Mutant) were packaged using HEK-293T cells and the viruses were used to infect the MDA-MB-231 cells as described previously^44^,^45^. Cells infected with the E-cadherin vectors were selected with 0.1 μg/ml puromyci. Stable expression was confirmed using polymerase chain reaction, and Western blotting.

### *In vivo* Tumor metastasis studies

Animal maintenance and experimental procedures were carried out in accordance with the US National Institute of Health Guidelines for Use of Experimental Animals and approved by the Medicine Animal Care Committee of Nanjing University (Nanjing, China). Experiments were carried out using female severe combined immune deficiency (SCID) mice (nu/nu; each 5 to 6 weeks old) as previously reported^46^. Mice were injected the cells via the lateral tail veinand sacrificed 8 weeks post-injection. Lungs were recovered and fixed with formalin, and the tumor foci on the surface of the left lobe were counted under a dissecting microscope, and then further processed for hematoxylin and eosin (H&E) staining and immunohistochemical staining for Ki-67 and E-cadherin.

### Statistical analysis

These above experiments were performed in triplicate, and each was repeated several times. The results are presented as the means ± SEM of at least three independent experiments. The differences were considered statistically significant at *P* < 0.05 using a Student’s *t*-test.

## Additional Information

**How to cite this article**: Pan, Y. *et al.* Slug-upregulated miR-221 promotes breast cancer progression through suppressing E-cadherin expression. *Sci. Rep.* **6**, 25798; doi: 10.1038/srep25798 (2016).

## Supplementary Material

Supplementary Information
