# Supplementary material for: Slug-upregulated miR-221 promotes breast cancer progression through suppressing E-cadherin expression
Source: Sci Rep. 2016 May 13;6:25798. doi: 10.1038/srep25798 (PMC4865839; doi:10.1038/srep25798)
Supplement: Supplementary Information [file srep25798-s1.pdf]

Slug-upregulated miR-221 promotes breast cancer progression through suppressing E-cadherin expression

Yi Pan[1,2+], Jing Li[1,2+], Yaqin Zhang[1,2,4+], Nan Wang[1,2], Hongwei Liang[1,2], Yuan Liu[3*], Chen-Yu Zhang[1,2*], Ke Zen[1,2,3*], and Hongwei Gu[1,2*]

[1]State Key Laboratory of Pharmaceutical Biotechnology, Nanjing Advanced Institute for Life Sciences (NAILS), Nanjing University, 22 Hankou Road, Nanjing, Jiangsu 210093, China; [2]Jiangsu Engineering Research Center for microRNA Biology and Biotechnology, Nanjing, Jiangsu 210093, China; [3]Center for Inflammation, Immunity and Infection & Department of Biology, Georgia State University, Atlanta, GA30302, USA. [4]Department of Biochemistry and Molecular Biology, School of Basic Medical Sciences, Nanjing Medical University, Nanjing, Jiangsu 210029, China;

Running title: Slug-promoted miR-221 targets E-cadherin

[+] These authors contributed equally to this work

[*] To whom correspondence may be addressed:

Hongwei Gu, PhD, Ke Zen, PhD and/or Chen-Yu Zhang, MD, PhD

State Key Laboratory of Pharmaceutical Biotechnology

Nanjing Advanced Institute for Life Sciences (NAILS)

22 Hankou Road, Nanjing, Jiangsu 210093, China

E-mail: hongweigu@nju.edu.cn, kzen@nju.edu.cn, and/or cyzhang@nju.edu.cn

Center for Inflammation, Immunity and Infection

Georgia State University

Atlanta, GA 30303, USA

E-mail: yliu@gsu.edu

Supplementary Table S1. Patients' Characteristics

Patients' characteristics				
Case No.	Clinical History	Gender	Age (years)	TNM Stage
BC #1	IDC	Female	46	II-III
BC #2	IDC	Female	50	III
BC #3	IDC	Female	45	II-III
BC #4	IDC	Female	48	III
BC #5	IDC	Female	56	II
BC #6	IDC	Female	49	II-III
BC #7	IDC	Female	60	III
BC #8	IDC	Female	47	III

Supplementary Table S2. List of miRNAs that are predicted to target E-cadherin ORF.

No.		Target-miRNA binding prediction	ΔG(kcal/mol)
1	has-miR-24	Target: 5' U A C G 3'<br>UUCC CU GGGCUGAGCU<br>AAGG GA CUUGACUCGG<br>miRNA: 3' GAC AC U 5'	-25.1
2	has-miR-107	Target: 5' C U CG C G 3'<br>AGCC CU GCG UGCUGCU<br>UCGG GA UGU ACGACGA<br>miRNA: 3' ACUA CA U 5'	-27.5
3	has-miR-133a	Target: 5' A GA CGGA A A 3'<br>AG GG GAAG GGACCAGG<br>UC CC CUUC CCUGGUUU<br>miRNA: 3' G GA AA C 5'	-26.6
4	has-miR-133b	Target: 5' A GA CGGA A A 3'<br>AG GG GAAG GGACCAGG<br>UC CC CUUC CCUGGUUU<br>miRNA: 3' A GA AA C 5'	-26.6
5	has-miR-202	Target: 5' C C 3'<br>CCCAU CUUUGUGCCUC<br>GGGUA GGGAUAUGGAG<br>miRNA: 3' AA C A 5'	-27.7
6	has-miR-210	Target: 5' A G A 3'<br>AGCU GCUG ACAUGUACGG<br>UCGG CGAC UGUGCGUGUC<br>miRNA: 3' AG AG 5'	-28.4
7	has-miR-218	Target: 5' A GAG C 3'<br>GGU GGGUUAAGCACAA<br>CCA UCUAGUUCGUGUU<br>miRNA: 3' UGUA A 5'	-27.4
8	has-miR-221	Target: 5' A A G UCAU C 3'<br>GGAAC CAG AG CAGUGUGGU<br>CUUUG GUC UC GUUACAUCG<br>miRNA: 3' G G U A 5'	-26.1

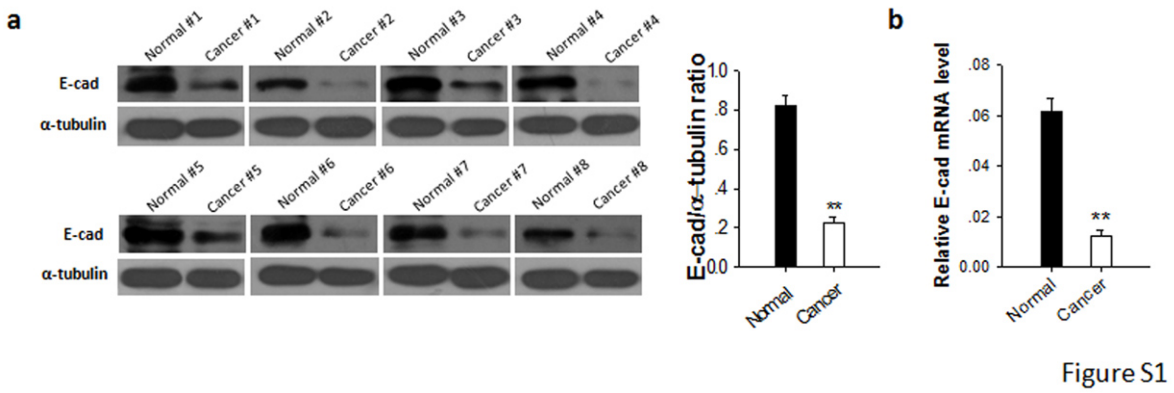

Supplementary Figure S1. Posttranscriptional regulation of E-cadherin in breast tumor tissues. (a-b) E-cadherin protein levels (a) and mRNA levels (b) in tumor tissue and normal tissue. Results are presented as the mean ± SEM (n=3). *, P<0.05. **, P<0.01.

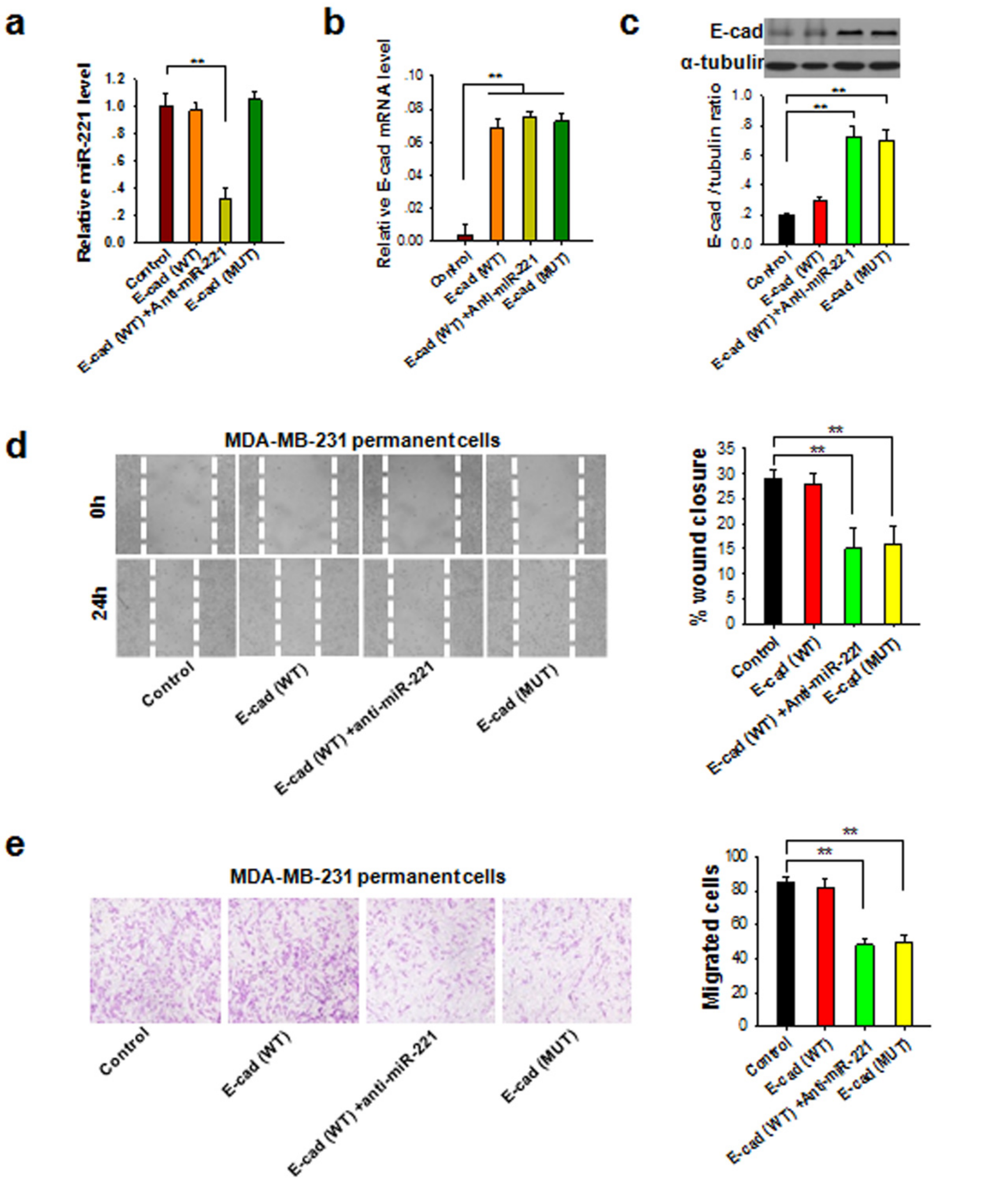

Figure S2

Supplementary Figure S2. Migration and invasion of MDA-MB-231 cells stably transfected with control Lentivirus (control) or Lentivirus expressing WT E-cadherin (E-cad (WT)), WT E-cadherin and anti-miR-221 (E-cad (WT) plus anti-miR-221), and MUT E-cadherin (E-cad (MUT)). (a) Relative miR-221 levels in four differently modified MDA-MB-231 cells. (b-c) Relative E-cadherin protein (b) and mRNA (c) levels in four differently modified MDA-MB-231 cells. Upper panel: representative image; Lower panel: quantitative analysis. (d-e) Migration (d) and invasion (e) of four differently modified MDA-MB-231 cells. Left panel: representative image; right panel: quantitative analysis. **, $P < 0.01$.
